# Review of Retinal Imaging Modalities for Hydroxychloroquine Retinopathy

**DOI:** 10.3390/diagnostics13101752

**Published:** 2023-05-16

**Authors:** Kai Xiong Cheong, Charles Jit Teng Ong, Priya R Chandrasekaran, Jinzhi Zhao, Kelvin Yi Chong Teo, Ranjana Mathur

**Affiliations:** 1 Singapore Eye Research Institute, Singapore National Eye Centre, 11 Third Hospital Avenue, Singapore 168751, Singapore; 2 Ophthalmology & Visual Sciences Academic Clinical Program (Eye ACP), Duke-NUS Medical School, Singapore 169857, Singapore

**Keywords:** HCQ retinopathy, toxicity, retinal imaging, optical coherence tomography, fundus autofluorescence, novel, retinal thickness, choroidal thickness, choroidal vascularity index, widefield imaging, en face imaging, minimum intensity analysis, artificial intelligence, near-infrared fundus autofluorescence, fluorescence lifetime imaging ophthalmoscopy, quantitative, optical coherence tomography angiography, adaptive optics, retromode imaging

## Abstract

This review provides an overview of conventional and novel retinal imaging modalities for hydroxychloroquine (HCQ) retinopathy. HCQ retinopathy is a form of toxic retinopathy resulting from HCQ use for a variety of autoimmune diseases, such as rheumatoid arthritis and systemic lupus erythematosus. Each imaging modality detects a different aspect of HCQ retinopathy and shows a unique complement of structural changes. Conventionally, spectral-domain optical coherence tomography (SD-OCT), which shows loss or attenuation of the outer retina and/or retinal pigment epithelium–Bruch’s membrane complex, and fundus autofluorescence (FAF), which shows parafoveal or pericentral abnormalities, are used to assess HCQ retinopathy. Additionally, several variations of OCT (retinal and choroidal thickness measurements, choroidal vascularity index, widefield OCT, en face imaging, minimum intensity analysis, and artificial intelligence techniques) and FAF techniques (quantitative FAF, near-infrared FAF, fluorescence lifetime imaging ophthalmoscopy, and widefield FAF) have been applied to assess HCQ retinopathy. Other novel retinal imaging techniques that are being studied for early detection of HCQ retinopathy include OCT angiography, multicolour imaging, adaptive optics, and retromode imaging, although further testing is required for validation.

## 1. Introduction

Hydroxychloroquine (HCQ; Plaquenil) is a synthetic medication that is used in the treatment of autoimmune and dermatological diseases. These include rheumatoid arthritis, systemic and discoid lupus erythematosus, Sjogren’s syndrome, and cutaneous vasculitis [1,2,3,4,5]. HCQ is a lipophilic and lysosomotropic drug. In its free base form, HCQ can pass through cell membranes and accumulate in lysosomes in the cytoplasm. The accumulation of HCQ increases the pH within the lysosomes, which contain hydrolytic enzymes that are normally activated by an acidic pH [6]. This results in the loss of lysosome stability. This in turn results in the impairment and inhibition of various functions, including antigen presentation, prostaglandin and cytokine production, Toll-like receptor signalling, and leucocyte activation. These effects allow HCQ to play its immunosuppressive, antiproliferative, antithrombotic, and photoprotective roles [7,8].

HCQ can cause toxicity to the eye, including the retina (retinopathy), anterior segment structures like the cornea (deposition of unmodified HCQ crystals in the epithelium), and, rarely, the ciliary body (disturbance of accommodation) [9,10,11]. Patients with HCQ retinopathy may not demonstrate any signs initially, but may progress to exhibit parafoveal or pericentral thinning of the outer retina, photoreceptor, and retinal pigment epithelium (RPE). This damage, which spares the foveal cones, produces the bull’s-eye configuration, and this is a late finding [12,13]. 

The pathophysiology of HCQ retinopathy is not completely understood. One proposed mechanism is the negative impact on the visual cycle through the inhibitory effects of HCQ and chloroquine of the organic anion transporting polypeptide 1A2 (OATP1A2), which is expressed in the RPE and is involved in all-trans-retinol recycling [14]. In the RPE, lysosomal activity may be affected by HCQ and this may impair phagocytosis of photoreceptor outer segments [15]. An alternative mechanism has been proposed in which the toxicity starts at the photoreceptors with secondary RPE degeneration. Lastly, some animal studies have also suggested that the earliest pathology in HCQ retinopathy begins in the retinal ganglion cells, with more advanced stages of degeneration progressing to the photoreceptors and RPE [16]. The accumulation of lipid complexes in the ganglion, bipolar, and glial cells has been postulated to be a triggering event for HCQ retinopathy [17,18]. In one study using rat models, chloroquine treatment induced lipidosis in the retina and subsequent electroretinogram assessments indicated continued degeneration of the photoreceptors despite remission of the lipidosis when chloroquine was withdrawn [17].

The prevalence of HCQ retinopathy is about 7.5%, but can increase to up to 20% to 50% after two decades of treatment [15]. The reported prevalence differs based on the dose, duration of use, and imaging modality used. The risk factors for HCQ retinopathy include a dose of greater than 5 mg/kg/day, concomitant use of tamoxifen, and impaired renal function of worse than 60 mL/min/1.73 m^2^ [19,20,21,22,23,24]. Underlying retinal disease may pose additional risks of toxicity [19,20,21,22,23,24].

HCQ retinopathy may result in progressive and irreversible loss of vision. Progression of damage can occur even after cessation of HCQ treatment, as HCQ can bind to melanin and accumulate in the RPE [25,26]. Thus, retinal screening is important. Several approaches to detect HCQ retinopathy have been investigated, but early detection has remained a challenge. 

Imaging is an essential aspect of the diagnosis and management of retinal conditions. The standard retinal imaging modalities used to assess HCQ retinopathy include optical coherence tomography (OCT) and fundus autofluorescence (FAF) [23,24]. Other novel retinal imaging techniques that are being studied include variations of OCT and FAF, OCT angiography (OCTA), multicolour imaging, adaptive optics (AO), and retromode imaging. Each imaging modality detects a different aspect of HCQ retinopathy and shows a unique complement of structural changes. Systematic reviews are useful for summarising large amounts of information and the review approach has been used previously to describe retinal imaging options for HCQ retinopathy [15,27,28]. Thus, this review aims to continue this work and to provide an up-to-date and comprehensive overview of standard and novel retinal imaging modalities for HCQ retinopathy.

## 2. Standard Retinal Imaging for HCQ Retinopathy

OCT and FAF are routinely available in outpatient settings, and are objective, fast, repeatable, and relatively cost-effective (Table 1) [29,30,31,32,33,34]. Appropriately, the updated Royal College of Ophthalmologists (RCOphth) and the American Academy of Ophthalmology (AAO) guidelines recommend the use of spectral-domain OCT (SD-OCT) and FAF for screening of HCQ retinopathy [23,24].

### 2.1. Optical Coherence Tomography

OCT is based on Michelson interferometry and typically uses near-infrared light [29,30,31]. The incident light is split with a beam splitter into the reference and sample beams. The backscattered light is measured with interferometry. As the reference beam path is known, the resultant interferometric signal can be used to calculate the sample beam, and therefore the depth profile of the sample at the selected location. This allows cross-sectional visualisation of the optical reflectivity of the tissue structure. 

Structural changes visible using OCT in HCQ retinopathy have been extensively described [23,35,36,37,38,39,40,41,42,43,44,45]. These structural changes may correspond with or precede other changes that are observed using perimetry, slit-lamp biomicroscopy, or electrophysiology testing. 

**Figure 1:**
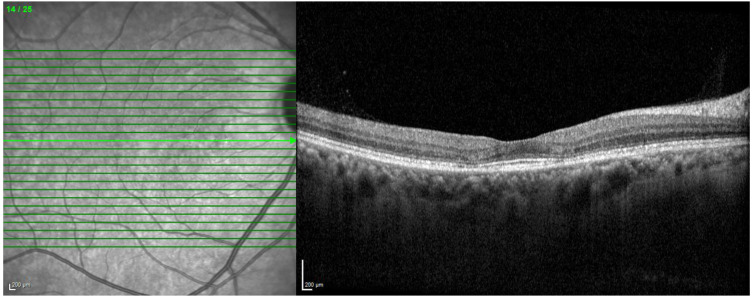
SD-OCT showing evident loss of the photoreceptors and outer retina in the parafoveal region, which causes the inner retinal layers to be displaced downwards around the fovea as the fovea is spared.

Early structural changes include attenuation and loss of the ellipsoid zone (EZ) in the parafoveal region and outer retinal loss with fovea sparing [45,46,47,48]. The macular subfield thickness maps may demonstrate parafoveal thinning even with minimal perimetric findings. Moderate changes are described as the “flying saucer” or “sombrero sign”, with more evident loss of the photoreceptors and outer retina, which causes the parafoveal inner retinal layers to be displaced downwards around the fovea as the fovea is spared [45]. (Figure 1). Whilst there is preservation of the RPE and external limiting membrane (ELM) in the advanced stages, the outer retina may demonstrate diffuse thinning beyond the fovea with marked outer retinal disorganisation and thinning/attenuation of the RPE–Bruch’s membrane complex [37]. 

Outer nuclear layer (ONL) thinning has also been reported to represent an early change in HCQ retinopathy. Lally et al., in a study of 30 eyes, characterised the structural changes in HCQ retinopathy using SD-OCT after drug cessation [42]. Findings before disruption of the parafoveal EZ included parafoveal ONL thinning in 100% of eyes, disruption and loss of the parafoveal interdigitation zone (IZ) in 88% of eyes, and reduced reflectivity or attenuation of the parafoveal EZ in 50% of eyes [42]. Whilst Lally et al. acknowledged that changes in reflectivity of the EZ may be difficult to identify as unremarkable OCT scans do exhibit variations in reflectivity, such variations should be homogeneous [42]. In contrast, more focal reflectivity changes of the parafoveal EZ are more likely to reflect true pathology [42].

IZ loss has been reported in other studies as well. Garrity et al. also reported OCT macular changes in a study of 10 patients with otherwise normal HVF, including parafoveal EZ and IZ loss/attenuation [49]. Several eyes progressed to advanced parafoveal outer retinal disruption and/or paracentral visual field defects [49].

**Figure 2:**
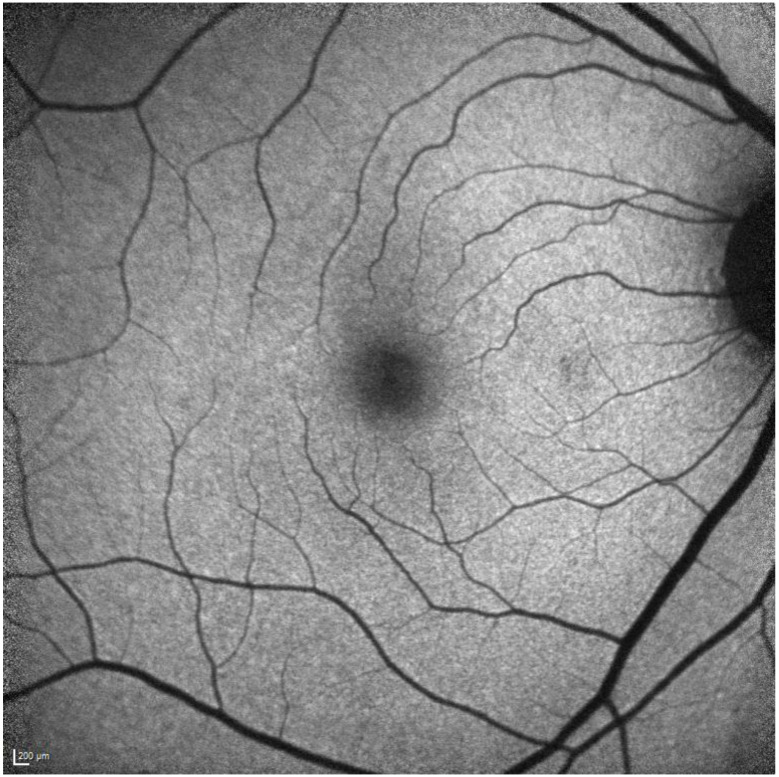
SD-OCT of an Asian patient showing similar outer retinal changes, including ellipsoid zone effacement, in the pericentral region.

Apart from parafoveal defects, pericentral defects may also develop (Figure 2). Melles et al. reported that a pericentral pattern of damage is particularly prevalent among Asians [43]. In their study, parafoveal was defined as 2° to 6° from the fovea, and pericentral was defined as ≥8° from the fovea [43]. The authors described in a study of 201 patients (18% Asian) that pericentral retinopathy alone was seen in 50% of Asian patients, but only in 2% of white patients [43]. Patients with the pericentral pattern consumed HCQ for a longer duration (19.5 versus 15.0 years, *p* < 0.01), took a larger cumulative dose (2186 g versus 1813 g, *p* = 0.02), and were diagnosed later compared with patients with the parafoveal pattern. This was thought to indicate that HCQ retinopathy is detected later for the pericentral pattern compared with the parafoveal pattern [43].

### 2.2. Fundus Autofluorescence

FAF makes use of the ability of endogenous fluorophores like lipofuscin to emit light when they are excited by suitable wavelengths [50,51,52]. It assesses the presence and distribution of the lipofuscin fluorescence within the RPE by using a 488 nm wavelength blue light [53]. HCQ has an affinity for RPE liposomes and interacts with lipofuscin, of which the bisretinoid N-retinylidene-N-retinylethanolamine (A2E) is a major component [19]. Hyperautofluorescence indicates excessive accumulation of lipofuscin in the RPE lysosomes, and in turn signals abnormal metabolism and/or phagocytosis of photoreceptor outer segments [27,52]. 

**Figure 3:**
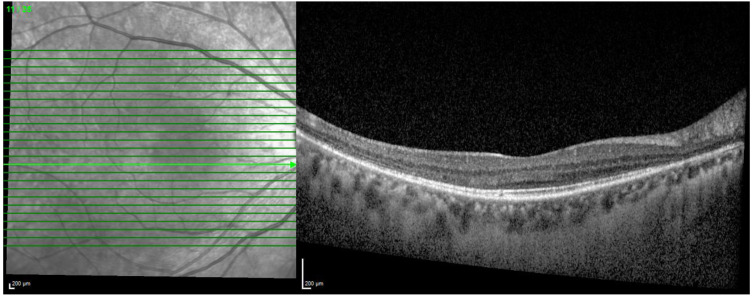
FAF showing parafoveal hyperautofluorescence corresponding to the changes in Figure 1.

**Figure 4:**
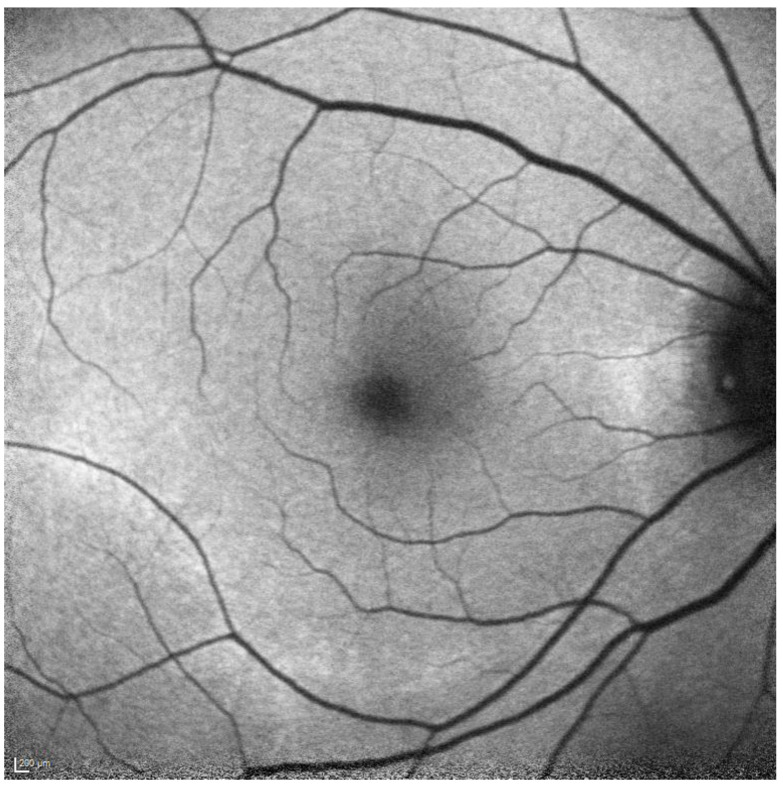
FAF showing pericentral hyperautofluorescence corresponding to the changes in Figure 2.

Parafoveal hyperautofluorescence with fovea sparing may be observed in the earlier stages of HCQ retinopathy (Figure 3) [54,55]. A bull’s-eye maculopathy pattern on FAF scans may even be observed before it is clinically seen in a slit-lamp biomicroscopy examination [56]. In more advanced stages, a mottled decrease in parafoveal autofluorescence may be detected due to RPE degeneration [15,55]. These changes have been correlated with concurrent abnormalities on SD-OCT scans. The distribution of fluorescence changes may similarly also be parafoveal or pericentral (Figure 4) [43].

### 2.3. Comparison of OCT and FAF

SD-OCT is generally considered to be more sensitive than FAF for the detection of HCQ retinopathy. A previous study of 57 patients by Cukras et al. reported that SD-OCT demonstrated better sensitivity and specificity in HCQ retinopathy detection compared with short-wavelength autofluorescence (SW-AF) [57]. Signs of HCQ retinopathy on SD-OCT and FAF scans were assessed and compared in affected versus unaffected patients, and were detected in 73.3% versus 9.1% for FAF, and in 84.2% versus 0.0% for EZ perifoveal loss on SD-OCT scans [57].

Jauregui et al. similarly reported in a study of 30 patients that SD-OCT was more sensitive in detecting HCQ retinopathy compared with SW-AF and near-infrared autofluorescence (NIR-AF) [58]. They described 10 patients who presented with early changes in the outer retinal layers (EZ attenuation and/or IZ continuity loss) in SD-OCT scans, yet with qualitatively normal SW-AF and NIR-AF scans [58]. Conversely, four patients presented with bull’s-eye maculopathy in SW-AF and NIR-AF scans with corresponding parafoveal outer retina disruption on SD-OCT scans [58]. There were no patients with SW-AF or NIR-AF abnormalities who had normal SD-OCT scans [58].

## 3. Novel Retinal Imaging for HCQ Retinopathy

Additionally, several variations of OCT (retinal and choroidal thickness measurements, choroidal vascularity index, widefield OCT, en face imaging, minimum intensity analysis, and artificial intelligence techniques) and FAF techniques (quantitative FAF, NIR-AF, fluorescence lifetime imaging ophthalmoscopy, and widefield FAF) have been applied for the assessment of HCQ retinopathy. Other novel retinal imaging techniques that are being studied for the early detection of HCQ retinopathy include OCTA, multicolour imaging, AO, and retromode imaging (Table 2).

### 3.1. Novel OCT Analysis

#### 3.1.1. Retinal Changes

There has been significant interest in discovering quantitative retinal parameters in HCQ retinopathy. Retinal thickness (RT) was amongst the first markers to be proposed. Retinal grids can be used to spatially divide the retina into regions. Mean aggregated RT measurements for each region can be manually or automatically measured by segmenting OCT images between the internal limiting membrane (ILM) and RPE–Bruch’s membrane complex. This measurement can be used in the diagnosis, prognostication, and treatment of chorioretinal diseases [59,60,61,62]. Characterisation of the RT in predefined areas helps to determine which regions of the retina are affected and to assess longitudinal changes. These data are readily obtainable using commercially available OCT devices.

Topographic analysis of whole RT has been explored by various groups. In a study of 1192 Korean patients with a history of HCQ treatment using the in-built software of a swept-source OCT system that automatically generated RT deviation maps, Kim et al. described five abnormal patterns in eyes with HCQ retinopathy, including pericentral (36.0%) or parafoveal (6.1%) ring, mixed-ring (34.2%), central island (13.2%), and whole macular thinning (10.5%) [63]. The presence of five or more contiguous red pixels showing one of these patterns in both eyes produced the highest diagnostic performance (discovery set: sensitivity and specificity of 98.2% and 89.1%; validation set: 100% and 87.5%, respectively) [63].

In addition to whole RT, current OCT analysis techniques can segment individual retinal layers. Changes in individual retinal layer thickness have been associated with HCQ retinopathy. Thinning of both the inner and outer retina has been described. Uslu et al. reported in a study of 30 patients that the sum thickness of the photoreceptor inner and outer segments was significantly lower in participants who took HCQ compared with that in the controls (*p* < 0.05) [64]. The RPE–Bruch’s membrane complex was significantly thicker, and the minimum and temporal-inferior macular ganglion cell–inner plexiform layer (GC-IPL) thicknesses were also significantly thinner in the patients using HCQ compared with the controls (*p* < 0.05, *p* = 0.04, and *p* = 0.03, respectively) [64].

##### Inner RT

Inner retinal thinning has been reported to be an early sign of HCQ retinopathy. In a study of 93 patients who took HCQ without signs of toxicity, Gil et al. reported that HCQ was associated with progressive parafoveal inner retinal thinning of the inner nuclear layer (INL) and ganglion cell layer (GCL) [65]. The parafoveal GCL thickness was inversely associated with cumulative dose (β = −0.239; *p* = 0.011) [65]. Similarly, Kan et al. demonstrated in another study of 90 patients macular GC-IPL thinning in patients who were treated with HCQ for at least five years [66]. The average, minimum, and sectorial macular GC-IPL thicknesses were significantly thinner in these patients compared with the controls, whilst the perimetry remained normal [66]. 

In a separate study of 46 patients by Bulut et al., the GC-IPL of patients who took HCQ was similarly thinner than that of controls in all regions (superior, superonasal, inferonasal, inferotemporal, and superotemporal) except inferiorly (*p* < 0.05) [67]. Additionally, there was a negative correlation between the mean GC-IPL thickness and cumulative HCQ dose (r = −0.371, *p* = 0.001) and the duration of use (r = −0.308, *p* = 0.006) [67]. 

Ulviye et al. also described significant inner retinal thinning at the parafoveal and perifoveal regions in the absence of fundus changes [18]. In this study of 15 patients who received HCQ treatment for five years, inner RT in the parafoveal and perifoveal area was significantly lower in the patients who received HCQ compared with that in the controls (*p* < 0.01 for perifoveal, *p* < 0.05 for parafoveal retinal measurements) [18]. However, significant thinning was demonstrated only in the full RT of the perifoveal area in the patients who received HCQ compared with the controls (*p* = 0.013) [18]. 

Other studies have also described RNFL thickness changes in patients treated with HCQ. In a study of 20 patients in which they were divided into Group I (HCQ/chloroquine toxicity with abnormal fundus), Group II (chronic exposure to HCQ/chloroquine without fundus changes), and Group III (controls), Pasadhika et al. reported that seven of eight eyes (88%) in Group I demonstrated peripapillary RNFL thinning in at least one quadrant, while none of the eyes in Groups II and III showed this finding [68]. Group I also demonstrated significant thinning of the inner, outer, and full-thickness retina (*p* < 0.001) and Group II had significant thinning only of the inner retina (*p* < 0.001) compared with Group III [68]. Polat et al., in another study of 49 patients, reported that inferior and nasal parafoveal RNFL, and temporal parafoveal GCL and IPL thicknesses were thinner, while the temporal parafoveal RPE was thicker in HCQ users [69].

##### Outer RT

Outer retinal changes occur in later stages of HCQ retinopathy. This has been reported by many studies. The ONL thickness is associated with HCQ retinopathy. Casado et al., in a study of 76 participants, reported that the ONL was significantly thinner in the HCQ retinopathy group compared with the controls at the nasal macula (*p* = 0.032); however, there were no significant differences in the GCL thickness between the groups [70].

In a study of 11 patients by Sisternes et al., while the topographic analysis showed that the inner retina was not associated with HCQ toxicity either between stages of retinopathy or after HCQ was stopped, HCQ toxicity localised to the parafoveal outer retina with a bull’s-eye maculopathy configuration in early to moderate stages of HCQ retinopathy damage [41]. However, this was followed by diffuse loss of photoreceptor cells across the macula as the damage became more severe [41].

##### Retinal Volume

Decrease of both inner and outer retinal volumes in patients who take HCQ has been reported. Modi et al. described in a study of 42 patients that the GCL (*p* = 0.01), IPL (*p* = 0.004), INL (*p* < 0.001), and outer plexiform layer (OPL) to RPE (*p* < 0.001) volumes were significantly reduced in HCQ retinopathy eyes compared with HCQ-exposed eyes [71]. Increasing disease severity correlated with increasing volume loss in the inner retina (2.27 mm in early disease versus 1.78 mm in advanced retinopathy, *p* = 0.02) [71].

Ugwuegbu et al. also reported significant outer retinal layer volumetric thinning compared with controls in HCQ retinopathy in a study of 14 patients. The ONL-EZ and EZ-RPE volumes and the parafoveal ONL-EZ thickness were significantly reduced in all HCQ retinopathy subgroups compared with the controls [38]. 

##### Longitudinal Changes in RT

Furthermore, longitudinal analysis of sequential inner and outer ETDRS ring macular thickness measurements can provide objective evidence of early structural changes in HCQ retinopathy before other clinical changes appear. Retinal thinning, which can precede the appearance of qualitative morphological changes on OCT scans, is helpful in HCQ retinopathy detection. In a study of 301 patients who received long-term HCQ treatment, Melles et al. reported a period of relatively linear rapid thinning with a loss of 3.75 ± 1.34 μm/year in 82 nonstable patients [72]. In contrast, the remaining 219 stable patients demonstrated a much less rapid thinning of 0.62 ± 0.45 μm/year [72]. Of the nonstable patients, 38 patients demonstrated conventional OCT or perimetric evidence of HCQ retinopathy, and, accordingly, greater thinning of 25.1 ± 6.2 μm compared with 15.7 ± 4.0 μm in those without HCQ retinopathy (*p* < 0.01) [72].

In a separate study of 144 patients by Godinho et al. that evaluated over a mean period of 38 months the longitudinal changes in retinal layer thickness of patients who were treated with HCQ without retinal toxicity, HCQ was associated with progressive thinning of the full retinal, inner retinal layer, and GCL thicknesses (foveolar: *p* = 0.040, *p* = 0.006; and *p* = 0.001, respectively; and paracentral: *p* = 0.006, *p* = 0.001; and *p* = 0.005, respectively) [73]. However, there were no changes in the outer retina [73]. Ugwuegbu et al. also reported progressive outer retinal thinning in a longitudinal analysis of patients with HCQ retinopathy [38].

#### 3.1.2. Choroidal Changes

The choroid is a complex three-dimensional vascular structure that is closely associated with the outer retina. As such, it has been implicated in the pathogenesis of many chorioretinal diseases. Accordingly, choroidal thickness (CT) and choroidal vascularity index (CVI) measurements have been reported to have the potential to diagnose, monitor, and prognosticate chorioretinal diseases [74,75,76,77]. CT is defined as the distance between the outermost edge of the RPE–Bruch’s membrane complex and the choroid–scleral interface. It is often measured at the foveal centre of the OCT B-scan, but can be measured at other points to provide a better global representation of CT. CVI, which is presented as a percentage, is defined as the ratio of the vascular area to the total choroidal area [78,79,80,81]. This is a quantitative measure of choroidal vascularity and requires processing of OCT images.

##### CT

Ahn et al. investigated 124 patients with systemic lupus erythematosus or rheumatoid arthritis following HCQ treatment and compared the CT between eyes with and without HCQ retinopathy [82]. They reported that CT was significantly decreased (*p* < 0.05) in the HCQ retinopathy group compared with the control group except at the temporal choroid 1.5mm away from the fovea [82]. While choriocapillaris thicknesses were significantly different in all choroidal locations between the groups, the medium-to-large vessel CT was not different at the nasal area 1.5 mm from the fovea nor at any temporal locations [82].

Polat et al., in a separate study of 49 patients, reported that subfoveal CT values were significantly reduced in patients who had used HCQ for fewer than five years (*p* = 0.042) and in patients who had used HCQ for five years or longer (*p* = 0.009) compared with the controls [69]. Parafoveally, CT was also reduced in the temporal sector, though not in the nasal sector, in patients who had used HCQ for five years or longer compared with the controls (*p* = 0.018) [69]. In a study of 47 patients, Halouani et al. also reported that the subfoveal and the mean CT were decreased in the advanced toxicity group compared with healthy controls (*p* < 0.001) [83].

##### CVI

Halouani et al. investigated choroidal involvement in HCQ retinopathy by assessing CVI, total choroidal area (TCA), luminal choroidal area (LCA), and stromal choroidal area (SCA) [83]. The CVI, TCA, LCA, and SCA were significantly lower in the advanced-stage HCQ toxicity group compared with the controls (*p* < 0.001, *p* < 0.00001, *p* < 0.0001, and *p* = 0.0094, respectively) [83].

#### 3.1.3. Widefield OCT

**Figure 5:**
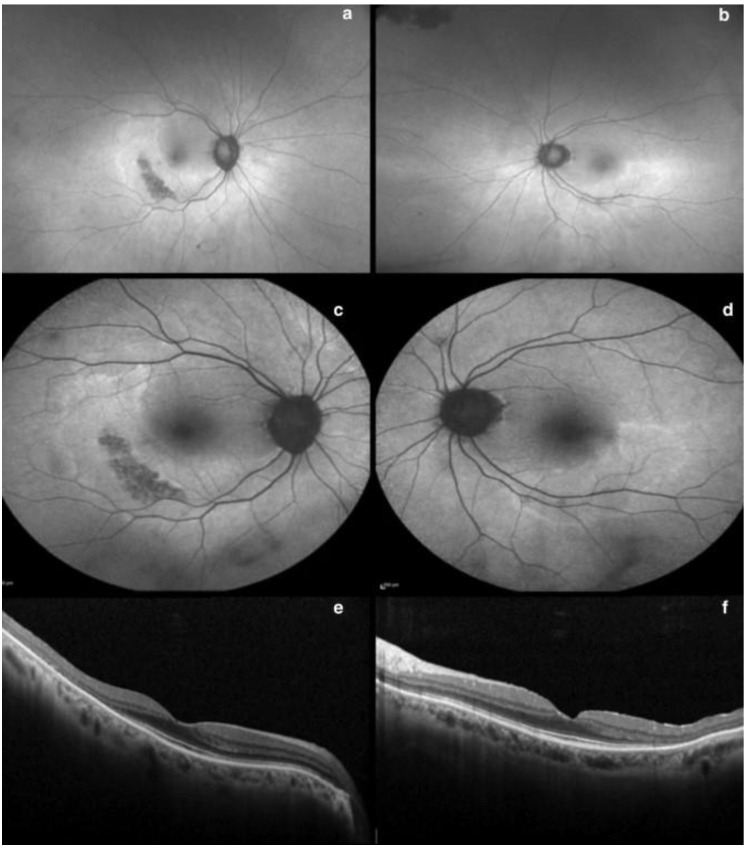
Optos ultra-widefield FAF (**a**,**b**) and Heidelberg FAF (**c**,**d**) scans demonstrating a more eccentric pericentral hyperautofluorescent ring corresponding to photoreceptor atrophy, sparing the fovea. SD-OCT displays bilateral and severe inner segment EZ loss in the temporal perifovea (**e**,**f**), which highlights the need for widefield retinal imaging [88]. [Reprinted from work by Corradetti et al. Source: Corradetti, G.; Violanti, S.; Au, A.; Sarraf, D. Wide field retinal imaging and the detection of drug associated retinal toxicity. *Int. J. Retin. Vitr.* **2019**, *5* (Suppl. S1), 26. https://doi.org/10.1186/s40942-019-0172-0. Distributed under the terms of the Creative Commons Attribution 4.0 International License (CC BY) (http://creativecommons.org/licenses/by/4.0/)].

The International Widefield Imaging Study group has defined widefield imaging as a single-capture image that is centred on the fovea, and which captures retinal anatomical features beyond the posterior pole, but posterior to the vortex vein ampulla in all four quadrants [84,85,86]. Widefield OCT is a useful option for the imaging of Asian patients, as retinal damage may extend beyond the macula (Figure 5) [43,87]. 

Ahn et al. explored the distribution of retinal changes in a study of 24 Asian patients with HCQ retinopathy and reported that the average minimum distance from the fovea to an area of photoreceptor defects was 1.84 ± 1.26 mm [89]. Only widefield line or volume scans could detect defects in the eyes [89]. 

#### 3.1.4. En Face OCT

**Figure 6:**
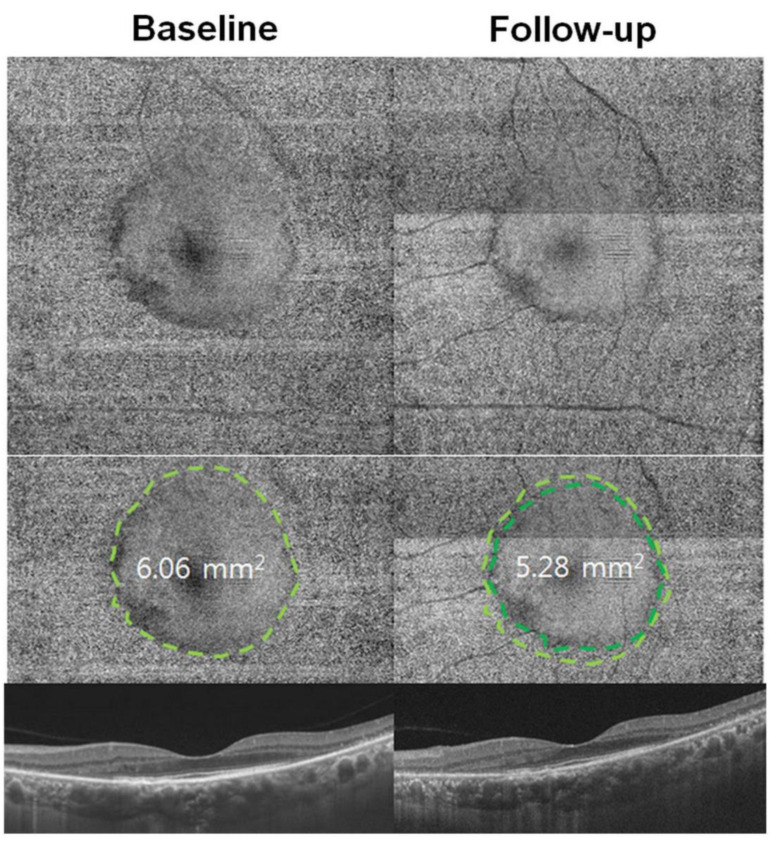
En face OCT in HCQ retinopathy demonstrates a smooth surface in the central area (inside the dashed line) on the areas with intact photoreceptors. In contrast, more granular reflectivity (outside the line) is noted in the pericentral area with retinal degenerative changes. The area with intact photoreceptors can be calculated and used as a quantitative measure of photoreceptor damage. The progression of photoreceptor damage is represented as a constricted ring at follow-up (10 months later). The quantitative measure of the area with intact photoreceptors decreased from 6.06 mm^2^ at baseline to 5.28 mm^2^ at the follow-up visit [28]. [Reprinted from work by Yusuf et al. Source: Yusuf, I.H.; Charbel Issa, P.; Ahn, S.J. Novel Imaging Techniques for Hydroxychloroquine Retinopathy. *Front. Med.* **2022**, *9*, 1026934. https://doi.org/10.3389/fmed.2022.1026934. Distributed under the terms of the Creative Commons Attribution 4.0 In-ternational License (CC BY) (http://creativecommons.org/licenses/by/4.0/)].

En face OCT is an emerging technique that combines SD-OCT with transverse confocal analysis, which generates transverse images and en face views of retinal and choroidal layers at a specified depth [90,91,92]. En face OCT can be used to assess structural changes in the coronal view and provide an extensive overview of pathological structures in a single image. Thus, it can be applied to characterisation of structural changes in HCQ retinopathy (Figure 6).

Ahn et al. investigated the application of en face OCT imaging in 31 patients with HCQ retinopathy and reported that HCQ retinopathy eyes exhibited a beaten-bronze appearance in the areas with photoreceptor defects, whereas those with intact photoreceptors showed areas with smooth surfaces, which were occasionally demarcated by hyporeflective margins [93]. Furthermore, en face OCT is also able to provide quantitative measurements, which can be objectively compared between visits. In the same study, Ahn et al. reported that the mean progression rate within the central 9 × 9 mm area was 0.59 mm^2^/year, ranging from −0.02 to 2.6 mm^2^/year [93].

Separately, de Sisternes et al. also demonstrated the utility of en face OCT by performing a pixel-by-pixel analysis of topographic SD-OCT data. They reported that HCQ toxicity primarily involved the outer retina [94]. En face thickness maps for the total retina, inner retina, and outer retina were generated by computing the axial differences between the corresponding segmented boundaries at each location in the horizontal-vertical plane [94].

#### 3.1.5. Minimum Intensity Analysis

Minimum intensity analysis involves the postacquisition analysis of each OCT A-scan to identify the lowest image intensity value in the region between the ILM and the RPE [95,96]. The ONL is the darkest layer in a normal retina and anchors the minimum intensity value. In HCQ retinopathy, the minimum intensity value is thought to increase due to changes in the optical reflectivity of the retinal layers.

In a study of 57 patients, Allahdina et al. reported that HCQ toxicity resulted in an increased ONL reflectivity [97]. They reported that the medians of the minimum intensity values in all subfields were significantly higher in the HCQ toxicity group compared with the unaffected group (*p* < 0.005 for all comparisons), with the largest difference found for the inner inferior subfield (*p* < 0.0001) [97]. The receiver operating characteristic analysis of the median minimum intensity values of the inner inferior subfields showed high sensitivity and specificity in the detection of toxicity with an area under curve (AUC) of 0.99 [97].

#### 3.1.6. Artificial Intelligence

Although OCT is an objective imaging modality, its current use still requires qualitative inspection of individual B-scans for EZ effacement and loss and/or thinning of other retina layers. This can be subjective, especially for early changes that are subtle and difficult to identify. This can be exacerbated by suboptimal image quality and varying levels of expertise among reviewing specialists. In this regard, an automatic detection and quantification of retinal changes on OCT would add objectivity and precision as a diagnostic aid for HCQ retinopathy screening.

Kalra et al. explored the feasibility of automated machine learning (ML)-based detection of HCQ retinopathy and prediction of progression to toxicity in eyes without pre-existing toxicity [98]. In this study of 388 participants, there were significant differences in partial EZ attenuation, EZ volume, ONL volume, and compartmental thicknesses, and clinical features, including HCQ daily dose, HCQ cumulative dose, and duration of therapy between the toxic and nontoxic groups [98]. Percentage area with partial EZ attenuation was the most discriminating feature. Using a random forest model, the toxicity detection model achieved a mean AUC of 0.97, sensitivity of 95%, and specificity of 91%, and the toxicity progression prediction model had a mean AUC of 0.89, with a sensitivity and specificity of 90% and 80%, respectively [98].

In a separate study of 85 patients using a mask-region-based convolutional neural network, De Silva et al. reported that the projection network that combined both horizontal and vertical scan analysis demonstrated the best overall performance (precision of 0.90 ± 0.09, recall of 0.88 ± 0.08, and F1 score of 0.89 ± 0.07) in detecting and quantifying EZ loss on SD-OCT images [99].

### 3.2. Novel FAF Analysis

#### 3.2.1. Quantitative Autofluorescence

Whilst FAF is a good and well-known technique for assessing HCQ retinopathy, FAF intensity can be quantified as well. The parafoveal autofluorescence in early HCQ retinopathy can be subtle, and quantitative autofluorescence (QAF) may increase the objectivity of assessment of this feature. QAF is an imaging modality that allows the measurement of autofluorescence following short-wavelength light (488 nm) excitation of bisretinoid fluorophores contained inside lipofuscin organelles [100,101,102]. QAF is based on SW-AF with a reference mirror installed to record and quantify the autofluorescence signal [100,101,102,103]. The intensity measurements are segmented by eight concentric segments at 7° to 9° eccentricity. The eight segments of the middle ring of three concentric rings (QAF_8_) have been widely accepted for QAF intensity analysis [32].

Increased QAF_8_ in HCQ retinopathy has been described. Greenstein et al. evaluated 31 patients and reported stronger QAF_8_ signals inferior to the fovea in patients who had bull’s-eye maculopathy due to HCQ [104]. As the signal was stronger in the inferior retina, it was postulated that the inferior retinal regions may experience more incident light and be more exposed to free-radical-mediated apoptosis secondary to increased light exposure and that HCQ may predispose the macula to light toxicity [104]. 

In a separate study of 39 patients, Parulli et al. similarly reported that the mean QAF values were higher in the inferior-temporal, inferior, and inferior-nasal sectors of the intermediate ring of QAF grid in patients on HCQ treatment compared with the untreated controls (all *p* < 0.05) [105]. The mean QAF_8_ was higher in patients treated with HCQ compared with the untreated controls (294.7 ± 65.3 versus 268.9 ± 57.5), though the difference was nonsignificant (*p* = 0.068) [105].

Reichel et al. also reported increased values of QAF as early as at six months after starting HCQ and chloroquine treatment in 44 patients without bull’s-eye maculopathy using a 97-segment grid instead of the conventional Delori patterned grid with 8 segments [106]. The mean QAF_97_ intensity was 278.1 ± 72.6 in patients without bull’s-eye maculopathy, compared with 235.4 ± 73.8 in the controls, *p* = 0.045 [106]. The QAF increase was reported to have begun early after starting treatment, remained high years after stopping treatment, and was not accompanied by changes apparent in the other imaging modalities including RT, except in cases with bull’s-eye maculopathy [106].

#### 3.2.2. Near-Infrared Autofluorescence

NIR-AF assesses the presence and distribution of melanin, which is an alternate endogenous fluorophore to lipofuscin, in the RPE and choroid [107,108,109]. Keilhauer et al. evaluated the origin of NIR-AF using scanning laser ophthalmoscopy and reported that it originates mostly from the melanin in the RPE and to a varying degree from the melanin in the choroid [110]. Elsner et al. previously reported that NIR wavelength light was better at delineating the pathology of subretinal structures than visible light [111]. 

**Figure 7:**
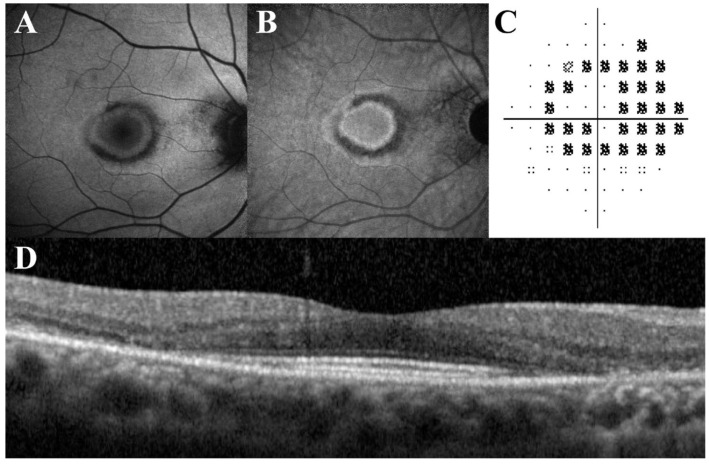
SW-AF (**A**) and NIR-AF (**B**) scans demonstrating normal patterns of autofluorescence centrally surrounded by a ring of hyperautofluorescence, which is surrounded by a region or ring of hypoautofluorescence in a bull's-eye pattern. Perimetry demonstrating a ring scotoma (**C**). On the SD-OCT (**D**) scan, the outer retina is significantly disrupted in a parafoveal manner, including loss of the EZ and IZ bands, with conservation of the fovea (“flying saucer”) [58]. [Reprinted from work by Jauregui et al. Source: Jauregui, R.; Parmann, R.; Nuzbrokh, Y.; Tsang, S.H.; Sparrow, J.R. Spectral-Domain Optical Coherence Tomography Is More Sensitive for Hydroxychloro-quine-Related Structural Abnormalities Than Short-Wavelength and Near-Infrared Autofluorescence. *Transl. Vis. Sci. Technol.* **2020**, *9*, 8. https://doi.org/10.1167/tvst.9.9.8. Distributed under the terms of the Creative Commons Attribution-NonCommercial-NoDerivatives 4.0 International License. (https://creativecommons.org/licenses/by-nc-nd/4.0/)].

It has been suggested that NIR-AF can help to detect HCQ toxicity before the onset of changes detectable using fundoscopy and OCT. Kellner et al. compared structural changes among NIR-AF, conventional lipofuscin-based FAF, SD-OCT, and multifocal electroretinogram scans in a study of eight patients. All patients with retinal structural abnormalities in the SD-OCT scans also showed NIR-AF changes [39]. In a subsequent study, Kellner et al. also used both SW-AF and NIR-AF in a study reporting the incidence of cystoid macular oedema and epiretinal membrane formation after cessation of HCQ/chloroquine treatment. Disease progression was also revealed with both imaging modalities [54]. In another study of 30 patients, Jauregui et al. reported that there were four patients (eight eyes) who presented with bull’s-eye maculopathy on SW-AF and NIR-AF images with corresponding outer retinal structures disrupted parafoveally on SD-OCT scans (Figure 7) [58]. Other authors have also similarly suggested that bull’s-eye maculopathy seen using near-infrared reflectance may represent early objective signs of HCQ retinopathy [112,113,114].

Nonetheless, the anatomical correlation of such NIR-AF changes is not entirely clear. While Chew et al. correlated the NIR-AF changes with IZ attenuation and outer-segment cone signal loss in reflectivity maps using AO imaging [114], Wong et al. commented that the demarcation line of the NIR bull’s-eye maculopathy lesion did not coincide with EZ changes on OCT scans [112]. 

#### 3.2.3. Fluorescence Lifetime Imaging Ophthalmoscopy

Fluorescence lifetime imaging ophthalmoscopy (FLIO) is a novel noninvasive imaging modality that is based on the principle of time-correlated single photon counting [115,116,117]. The fluorescence lifetime represents the time span that an endogenous fluorophore such as lipofuscin spends at a higher energy level following an excitation with a blue laser before returning to its ground level by releasing a photon with a longer emission wavelength [115,116,117]. Photons are detected in two separate spectral channels (a short spectral channel (SSC) and a long spectral channel (LSC)) [115,116,117].

Earlier detection of HCQ retinopathy appears to be feasible with FLIO, although the clinical benefits of FLIO have not been fully evaluated and the imaging modality is not yet widely available. In a study of 12 patients, Sauer et al. reported that HCQ toxicity demonstrated significantly prolonged FLIO lifetimes in regions of damage, typically in a bull’s-eye configuration that corresponded with toxic lesions in the retina compared with unaffected retinas (SSC: 400 ps versus 294 ps; and LSC: 404 ps versus 316 ps; *p* both <0.001) [118]. In contrast to age-matched controls, clinically normal patients who were assessed to be at high risk of developing HCQ toxicity also demonstrated prolonged FLIO lifetimes in the parafoveal region. In another study of 21 patients by Solberg et al., prolonged parafoveal ring-shaped or oval areas of increased mean FLIO lifetimes compared with the controls (SSC: 374 ps versus 313 ps, *p* = 0.0001) were also reported [119]. 

#### 3.2.4. Widefield FAF

**Figure 8:**
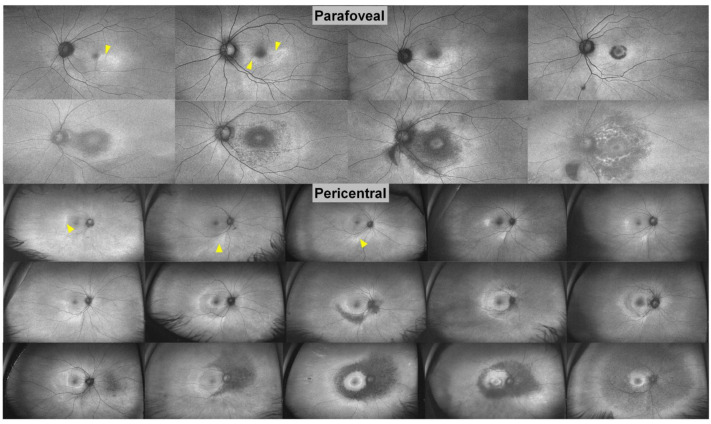
Ultra-widefield FAF in parafoveal and pericentral HCQ retinopathy. The images are placed in order of increasing severity and retinal damage based on the extent of hyper- and hypoautofluorescence, from temporal or inferior patchy hyperautofluorescence (arrowheads) to extensive hypoautofluorescence [28]. [Reprinted from work by Yusuf et al. Source: Yusuf, I.H.; Charbel Issa, P.; Ahn, S. J. Novel imaging techniques for hydroxychloroquine retinopathy. *Front. Med.* **2022**, *9*, 1026934. https://doi.org/10.3389/fmed.2022.1026934. Distributed under the terms of the Creative Commons Attribution 4.0 In-ternational License (CC BY) (http://creativecommons.org/licenses/by/4.0/)].

Similar to widefield OCT, widefield FAF is also useful for imaging of Asian patients, as retinal damage may extend beyond the macula [43,87]. Ahn et al. compared ultra-widefield FAF with conventional FAF in a study of 29 patients with HCQ retinopathy [120]. Ultra-widefield is defined as a single-capture image that is centred on the fovea, and which captures retinal anatomical features anterior to the vortex vein ampulla in all four quadrants [84,85,86]. It was noted that while abnormal FAF findings were noted in the retinal periphery outside the conventional FAF field of view as hypoautofluorescent and hyperautofluorescent lesions in 70.7% of the eyes, there was conversely no peripheral extension beyond the coverage of the ultra-widefield FAF [120]. This highlights the utility of ultra-widefield FAF in evaluating the extent of peripheral changes in HCQ retinopathy within a single image (Figure 8) [120].

### 3.3. OCT Angiography

OCTA is a noninvasive and dye-free technique that yields high-resolution and depth-resolved images of the retinal vasculature without intravenous dye administration [121,122,123]. OCTA uses the motion contrast of moving erythrocytes and compares decorrelation signals between successive OCT B-scans that are taken at the same tissue location in dense volumetric scans. As axial bulk motion is removed, motion between B-scans represents erythrocyte movement. OCTA yields quantitative measurements including vessel and perfusion density of the superficial (SCP) and deep capillary plexus (DCP). En face images of vascular changes can be correlated with corresponding structural changes on OCT. An important limitation of many commercially available OCTA devices is the field of view [124]. Nonetheless, as widefield devices enter the market, this limitation will be overcome.

The evidence for the use of OCTA is mixed. Whilst some studies have highlighted the utility of OCTA to detect vessel density changes in early stages of HCQ retinopathy even when clinical changes are not apparent, others have suggested it may not be suitable. 

**Figure 9:**
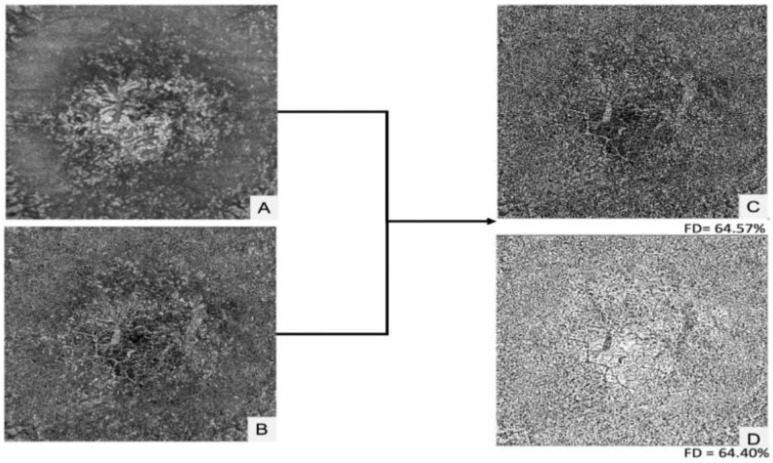
Choriocapillaris analysis using the Phansalkar local thresholding method in a patient’s eye with HCQ retinopathy. Flow deficit (FD) quantification (**A**–**D**) suggests that the choriocapillaris is involved in HCQ retinopathy [126]. [Reprinted from work by Halouani et al. Source: Halouani, S.; Le, H. M.; Cohen, S. Y.; Terkmane, N.; Herda, N.; Souied, E. H.; Miere, A. Choriocapillaris Flow Deficits Quantification in Hydroxychloroquine Retinopathy Using Swept-Source Optical Coherence Tomography Angiography. *J. Pers. Med.* **2022**, *12*, 1445. https://doi.org/10.3390/jpm12091445. Distributed under the terms of the Creative Commons Attribution 4.0 International License (CC BY) (http://creativecommons.org/licenses/by/4.0/)].

In studies which suggested that OCTA is useful, HCQ retinopathy generally manifested as damage to the DCP, choriocapillaris, and, less commonly, the SCP and/or foveal avascular zone (FAZ) enlargement (Figure 9). Akhlaghi et al. investigated OCTA parameters in 61 patients with HCQ retinopathy who were diagnosed using multifocal electroretinograms and reported that the mean vessel density in the DCP in the whole image, superior hemifield, inferior hemifield, and perifoveal area was lower compared with the control group (*p* all <  0.05) [125]. There were no differences in vessel density of the SCP between groups [125].

In another study by Ahn et al. of 20 patients with systemic lupus erythematosus or rheumatoid arthritis who were diagnosed to have HCQ retinopathy, OCTA demonstrated signal void areas on the choriocapillaris corresponding to RPE defects on swept-source OCT B-scans, which were more remarkable in those with advanced disease [40]. The authors further suggested an association between choriocapillaris involvement and disease progression after HCQ cessation [40].

Forte et al. evaluated swept-source OCTA in a study of 10 patients who were treated with HCQ for more than five years, and described that in comparison with the control group, the vessel densities in the central 1 mm sector of the choriocapillaris and in the central 1mm sector and nasal and temporal subfields of the DCP were decreased [127]. There was also an increased FAZ in the capillary plexuses, a greater frequency of choriocapillaris flow voids, and a reduced foveal CT (*p* < 0.05). They further suggested that choriocapillaris flow abnormalities may precede RPE disruption [127]. 

Studies have further reported greater microvascular damage associated with HCQ use of a longer duration and greater cumulative dose. In a larger study of 60 patients, Bulut et al. reported that patients in the high-risk group (five years of HCQ use or more) demonstrated greater vascular density loss and a wider FAZ, as well as decreased retinal and choroidal flow rates (*p* all < 0.05), compared with the low-risk group (less than five years of HCQ use) [67].

Ozek et al., in a study of 40 patients, also compared the vessel densities of participants who had taken HCQ for more than five years (Group 1), participants who had taken HCQ for fewer than five years (Group 2), and a healthy control group (Group 3) [128]. Ozek et al. described that the parafoveal deep temporal and deep hemi-inferior vascular plexus density was reduced in Group 1 compared with Group 3 (*p* = 0.041 and *p* = 0.046, respectively) [128].

Interestingly, some studies have reported that HCQ may play a protective role in preserving microvascular structures. The mechanism is unclear. Mihailovic et al. evaluated the vessel density in 19 patients with systemic lupus erythematosus who were treated with HCQ [129]. The study group was divided into patients who had used HCQ for more than five years (high-risk group) and fewer than five years (low-risk group). While vessel density in the SCP was significantly reduced in both groups compared with that in the control group (*p* < 0.001, and *p* = 0.001, respectively) and in the high-risk group compared with the low-risk group (*p* = 0.007), the low-risk group exhibited a significant positive correlation between cumulative HCQ dose and vessel density (*p* = 0.035) [129]. The group hypothesised that HCQ may exert a protective effect only in the early stages of the disease [129].

Conigliaro et al. also described in a cohort of 20 patients with systemic lupus erythematosus without retinopathy changes that there was a positive correlation between HCQ cumulative dose and both superficial and deep parafoveal density (r = 0.4, *p* = 0.009; and r = 0.3, *p* = 0.04, respectively) [130]. While this study also suggested a protective effect of HCQ on vessel density, it did not distinguish between patients with different durations of HCQ treatment.

On the other hand, Esser et al. reported that vessel density was not decreased in a rheumatoid arthritis group compared with the control group (*p* > 0.05) [131]. They concluded that OCTA may not be suitable for early detection of HCQ retinopathy and that the focus should be on intensive and sequential OCT analysis for early detection of HCQ retinopathy [131].

### 3.4. Multicolour Imaging

Multicolour imaging is a relatively new technology that captures three simultaneous reflectance images using monochromatic infrared (815 nm), green (518 nm), and blue (486 nm) light [132,133,134]. The infrared image shows the outer retina and choroid, the green image shows blood vessels, haemorrhages, and exudates, and the blue image shows the inner retina and vitreoretinal interface. These three images are combined to create a high-resolution multicolour image, which is noninvasive and can be taken through a small pupil. Compared with conventional white light colour fundus photography, it provides better topographical information, such as localised retinal thickening or thinning [135,136]. 

**Figure 10:**
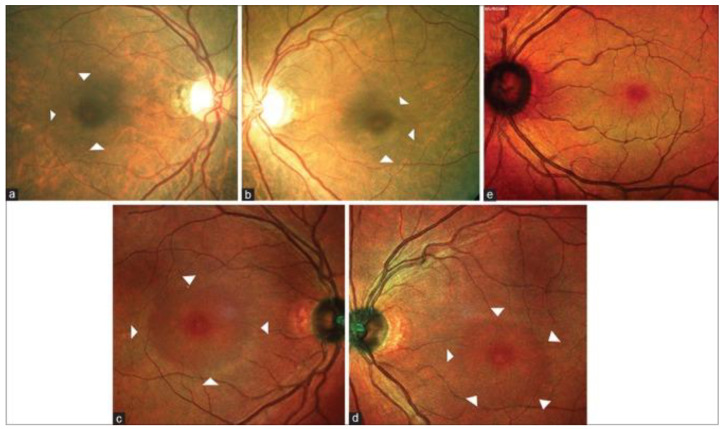
Colour fundus photography of the right and left eyes showing arcuate zone of hypopigmentation (white arrow heads) superior, temporal, and inferior to fovea (**a**,**b**). Multicolour imaging of the right and left eyes showing rings of darker hue around fovea (white arrow heads) corresponding to the arcuate hypopigmentation seen on colour fundus photography and extending beyond (**c**,**d**). Multicolour imaging image of the left eye of a normal individual showing the deep pink fovea centre surrounded by a greenish hue that is missing in the eye with HCQ retinopathy (**e**) [137]. [Reprinted from work by Saurabh et al. Source: Saurabh, K.; Roy, R.; Thomas, N.R.; Chowdhury, M. Multimodal Imaging Characteristics of Hydroxychloroquine Retinopathy. *Indian J. Ophthalmol.* **2018**, *66*, 324–327. https://doi.org/10.4103/ijo.IJO_787_17. Distributed under the terms of the Creative Commons Attribu-tion-NonCommercial-ShareAlike 3.0 License (CC BY-NC-SA 3.0) (https://creativecommons.org/licenses/by-nc-sa/3.0/)].

In a case report of a patient who had taken HCQ for approximately six years, Saurabh et al. reported that the composite multicolour image demonstrated a circumscribed perifoveal arcuate area of darker hue sparing the fovea bilaterally (Figure 10) [137]. The multicolour image changes corresponded to the zone of retinal thinning on SD-OCT scans. The infrared reflectance image demonstrated speckled hyperreflectance at the central macula with a surrounding arcuate zone of hyporeflectance, which corresponded with the zone of outer retinal thinning on SD-OCT scans [137]. 

### 3.5. Adaptive Optics

**Figure 11:**
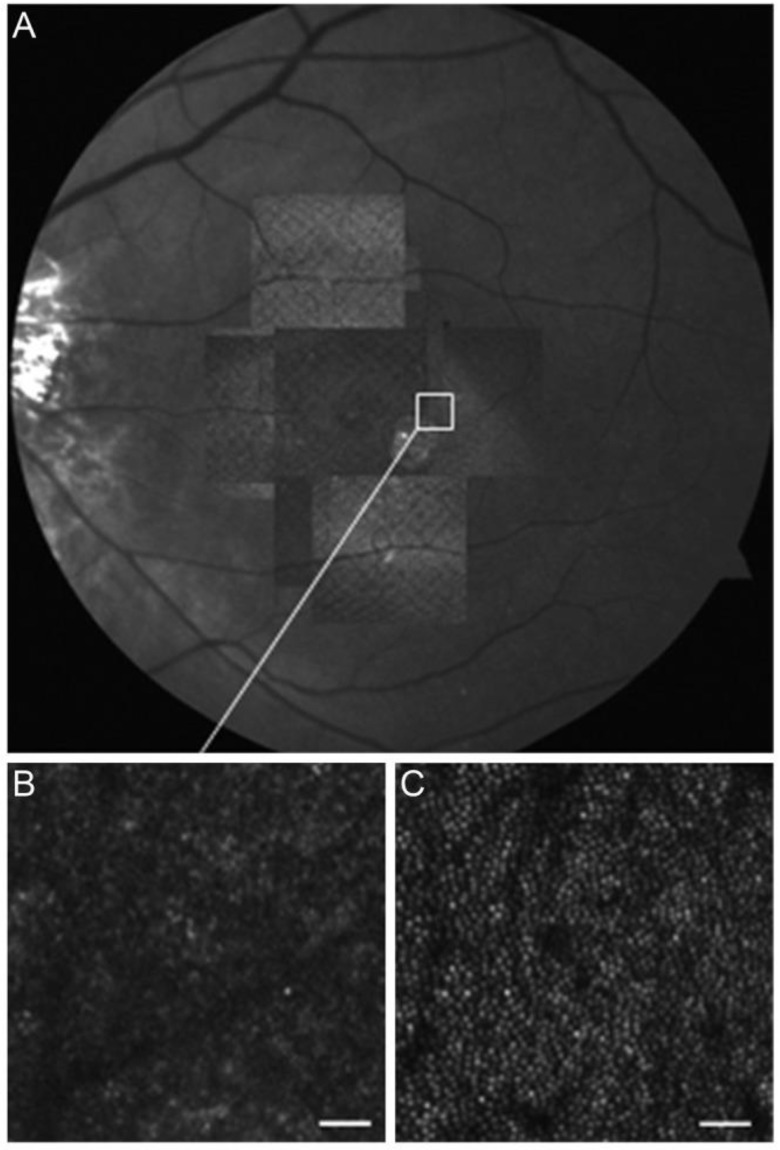
AO scanning laser ophthalmoscope (AO-SLO) montage of the left eye (**A**) matched with the corresponding red-free image. Magnified AO-SLO images are also shown (**B**,**C**). (**B**) shows the area indicated by the white box on the montage and it demonstrates disruptions in the cone mosaic, where cones are missing or lost. The cones are also asymmetrical in shape and size with variable brightness. (**C**) shows an age-matched normal retina in the same location without the structural changes seen in (**B**) [142]. [Reprinted from work by Bae et al. Source: Bae, E. J.; Kim, K. R.; Tsang, S. H.; Park, S. P.; Chang, S. Retinal damage in chloroquine maculopathy, revealed by high resolution imaging: a case report utilizing adaptive optics scanning laser ophthalmoscopy. *Korean J. Ophthalmol.* **2014**, *28*, 100–107. https://doi.org/10.3341/kjo.2014.28.1.100. Distributed under the terms of the Creative Commons Attribu-tion-NonCommercial 3.0 Unported License (CC BY-NC 3.0) (http://creativecommons.org/licenses/by-nc/3.0/)].

AO uses active optical elements to compensate for optical aberrations between the object and the camera. Applied to the human eye, AO allows direct visualisation of individual rod and cone photoreceptor mosaic, RPE cells, and white blood cells [138,139,140]. This technology allows for optical resolutions of less than 2 μm, which is sufficient for measurements of cellular and subcellular details of normal retina to be made; thus, AO has the potential to detect very early signs of HCQ retinopathy (Figure 11) [141].

In a pilot study of 23 patients, Debellemanièree et al. reported that parafoveal cone metric changes may represent the earliest sign of HCQ macular toxicity. Moderate cone loss and cone spacing were associated with increasing HCQ dose (linear regression r^2^ = 0.23, *p* = 0.018; and r^2^ = 0.17, *p* = 0.008, respectively) [143].

Babeau et al. similarly described in another study of 38 patients that age- and gender-adjusted cone density in the inferior quadrant was significantly lower in patients who took a daily dose of ≥6.5 mg/kg/day compared with those who took <6.5 mg/kg/day (22,339.23 versus 20,480.93, *p* = 0.035) [144]. The mean cone density in the inferior quadrant was lower in patients who took a cumulative dose of ≥1000 g compared with those who took <1000 g (20,593.85 versus 22,365.04, *p* < 0.008) [144]. 

Separately, Stepien et al. described in a study of four patients that there was a loss of cone mosaic in the perifoveal region that corresponded with the OCT findings, and further demonstrated EZ loss with preservation of the RPE and ELM, showing a “sinking hole defect” [47]. 

However, in a separate prospective study of patients who were treated with HCQ for a minimum of two years, Ueda-Consolvo et al. reported that there was no significant decrease in cone density in the first two years of HCQ use [145].

### 3.6. Retromode Imaging

Scanning laser ophthalmoscopy in the retromode illuminates the fundus with an infrared laser and collects the backscattered light that is reflected from the retina, choroid, and sclera. Using a laterally deviated confocal aperture with a central stop, which creates pseudo-three-dimensional images with shadows and enhances the contrast of the lesion, the outer retina and RPE can be better visualised. It can be used in the diagnosis and management of ocular diseases, including HCQ retinopathy, age-related macular degeneration, and central serous chorioretinopathy [146,147,148].

Ahn et al. conducted a study of 247 patients regarding the use of retromode imaging to detect HCQ retinopathy [146]. Retromode imaging had a 100% sensitivity in HCQ retinopathy detection and all patients with HCQ retinopathy demonstrated parafoveal or pericentral ring-shaped or round areas of decreased reflectance with prominent deep choroidal vessels [146]. The specificity of retromode imaging was 73.0% and 76.4% in the control group which took HCQ and a separate control group which did not take HCQ, respectively [146]. It was noted that while retromode imaging had a higher sensitivity for the detection of photoreceptor defects compared with FAF, particularly in early HCQ retinopathy, it was unable to discriminate between photoreceptor and RPE defects, unlike FAF [146].

## 4. Conclusions

This review provides a detailed overview of standard and novel retinal imaging modalities for HCQ retinopathy. Retinal imaging is important as HCQ retinopathy may result in progressive and irreversible loss of vision, and progression of damage can occur even after cessation of HCQ treatment. Recognition of the structural changes using retinal imaging is likely to lead to a better appreciation of the pathophysiology of HCQ retinopathy, and helps in the diagnosis, treatment, and prognostication of HCQ retinopathy.

In general, OCT and FAF are the main imaging modalities used to detect HCQ retinopathy because they are accurate, rapid, and noninvasive means of generating structural information regarding the disease, and they are generally available in most ophthalmology practices. Other novel retinal imaging techniques that are discussed in this review include novel analyses using OCT and FAF, OCTA, multicolour imaging, AO, and retromode imaging. However, further testing is required for validation of their clinical significance. Longitudinal data are also lacking.

Despite the ubiquitous use of retinal imaging modalities such as SD-OCT and FAF, there remains very little agreement regarding the diagnostic performance of the various imaging modalities for the detection of HCQ retinopathy. In addition, existing screening processes rely on the recognition of structural changes on OCT and FAF scans, which can be subjective, especially in early HCQ retinopathy. In this regard, a multimodal imaging strategy that synergistically combines the various morphofunctional characteristics of HCQ retinopathy would be ideal. There should be more studies done on the cost-effectiveness of these studies as well, and additional modalities should be incorporated into existing screening processes to enable personalised care.

It is difficult to compare studies as different studies have considered different patient populations (of varying age, ethnicity, disease, etc.), duration and dose of HCQ use, and imaging modalities. The discordance is evident, as various studies have reported in favour of different modalities. All studies in this review were hospital-based in design and most had very small study population sizes, so this limits the external validity of the studies to some extent. A more objective definition of HCQ retinopathy that utilises known structural changes from these imaging modalities may aid the diagnosis, treatment, and prognostication of HCQ retinopathy.

On a separate note, considering the plethora of structural changes of HCQ retinopathy that can be observed using the various imaging modalities, a relevant question would be when to discontinue or reduce the HCQ dose. Relevant to this question is the joint statement from the American College of Rheumatology, American Academy of Dermatology, Rheumatologic Dermatology Society, and AAO regarding HCQ use in HCQ retinopathy that was published in 2020 [149]. It is important for medical practitioners to not only consider the risk and extent of visual loss in HCQ retinopathy, but also to understand and appreciate the value and clinical indication of starting HCQ in the first place. In this regard, HCQ should not be discontinued without proper consideration [149]. HCQ retinopathy takes time (years) to develop and there is time for subtle and suspicious findings to be confirmed with multimodal imaging and for retinal consultations to confirm the diagnosis [149]. Suspicious findings should be discussed with the patients and prescribing physicians, and HCQ should not be stopped prematurely until the diagnosis is confirmed through shared decision making that involves all stakeholders [149].

The RCOphth recommends that HCQ treatment should continue until the outcome of electrophysiology is known, to decrease the risk of inappropriate discontinuation of HCQ treatment [23]. Similarly, it recommends that recommendations to discontinue HCQ, should there be a valid cause, be made to the prescribing physicians to facilitate discussion among the stakeholders regarding drug alternatives and the risk of HCQ discontinuation [23]. It explicitly states that it would be inappropriate for ophthalmologists to stop HCQ treatment [23].

In conclusion, there have been rapid advancements made in retinal imaging for HCQ retinopathy. The novel retinal imaging modalities are rapid, noninvasive, easy to use, and increasingly available in clinical settings. While all of them show promise, larger studies and longitudinal data are required to allow a better understanding of the pathophysiology of HCQ retinopathy and to allow more accurate and cost-effective diagnosis, management, and prognostication of HCQ retinopathy. Future screening programmes should incorporate these developments as well.

## Figures and Tables

**Table 1:** Standard retinal imaging modalities for hydroxychloroquine (HCQ) retinopathy.

Modality	Findings	Advantages and Disadvantages
**Optical coherence tomography**	▪ Attenuation and loss of ellipsoid zone ▪ Outer retinal loss with fovea sparing (flying saucer or sombrero sign) ▪ Marked outer retinal disorganisation and thinning/attenuation of retinal pigment epithelium–Bruch’s membrane complex ▪ Outer nuclear layer thinning ▪ Loss of interdigitation zone ▪ Pericentral defects, as opposed to parafoveal defects, in Asians	▪ Rapid and accurate cross-sectional imaging of the retina ▪ More sensitive and specific in detection of HCQ retinopathy compared with FAF ▪ Early changes may be subjective and difficult to identify
**Fundus autofluorescence**	▪ Parafoveal or pericentral hyper- and/or hypoautofluorescence with fovea sparing ▪ Bull’s-eye maculopathy pattern	▪ Allows a quick view of structural changes at the posterior pole in a single image ▪ Changes can be correlated with concurrent abnormalities on SD-OCT scans ▪ Changes may not be detected as early as in SD-OCT

**Table 2:** Novel retinal imaging modalities for hydroxychloroquine (HCQ) retinopathy.

Modality	Findings	Advantages and Disadvantages
**Optical coherence tomography**		
Retinal thickness	▪ Whole retinal thickness thinning ▪ Inner retinal thinning of retinal nerve fibre layer, ganglion cell layer, inner plexiform, and/or inner nuclear layer as early signs of HCQ retinopathy ▪ Outer retinal thinning of outer nuclear layer ▪ Precede classic morphologic changes	▪ May allow earlier detection of HCQ retinopathy ▪ Allows quantitative analysis
Retinal volume	▪ Decrease of inner and/or outer retinal volume	▪ May allow earlier detection of HCQ retinopathy ▪ Allows quantitative analysis
Choroidal thickness	▪ Decrease in choroidal thickness	▪ May allow earlier detection of HCQ retinopathy ▪ Allows quantitative analysis
Choroidal vascularity index	▪ Decrease in choroidal vascularity index	▪ May allow earlier detection of HCQ retinopathy ▪ Allows quantitative analysis
Widefield OCT	▪ Helpful to detect pericentral and more peripheral defects	▪ May allow earlier detection of HCQ retinopathy ▪ Assesses structural changes of HCQ retinopathy in the peripheral retina
En face imaging	▪ Beaten-bronze appearance in areas with photoreceptor defects	▪ Assesses structural changes of HCQ retinopathy in the coronal plane and provides an extensive overview of pathological structural changes in the macula in a single image ▪ May allow earlier detection of HCQ retinopathy ▪ Allows quantitative analysis
Minimum intensity analysis	▪ Increased reflectivity	▪ May allow earlier detection of HCQ retinopathy ▪ Allows quantitative analysis
**Fundus autofluorescence**		
Quantitative FAF	▪ Increased quantitative autofluorescence intensity	▪ May allow earlier detection of HCQ retinopathy ▪ Allows quantitative analysis
Near-infrared autofluorescence	▪ Parafoveal or pericentral hyper- and/or hypoautofluorescence with fovea sparing ▪ Bull’s-eye maculopathy pattern	▪ May allow earlier detection of HCQ retinopathy ▪ Alternate endogenous fluorophore to lipofuscin
Fluorescence lifetime imaging ophthalmoscopy	▪ Prolonged fluorescence lifetime imaging ophthalmoscopy lifetimes	▪ May allow earlier detection of HCQ retinopathy ▪ Allows quantitative analysis
Widefield FAF	▪ Helpful to detect pericentral and more peripheral defects	▪ May allow earlier detection of HCQ retinopathy ▪ Assesses structural changes of HCQ retinopathy in the peripheral retina
**Optical coherence tomography angiography**	▪ Decreased vascular density of deep and superficial capillary plexuses ▪ Signal voids in choriocapillaris ▪ Increased foveal avascular zone size	▪ May allow earlier detection of HCQ retinopathy ▪ Allows quantitative analysis ▪ May have limited field of view
**Multicolour imaging**	▪ Speckled hyperreflectance at central macula with surrounding arcuate zone of hyporeflectance on infrared reflectance image that corresponds with outer retinal thinning	▪ May allow earlier detection of HCQ retinopathy ▪ Three reflectance images of the retina that form a composite image allow analysis of structural changes of HCQ retinopathy at various levels within the retina
**Adaptive optics**	▪ Loss of cone mosaic and density	▪ Earlier detection of photoreceptor damage
**Retromode imaging**	▪ Parafoveal or pericentral ring-shaped or round area of decreased reflectance with prominent deep choroidal vessels	▪ May have a higher sensitivity to detect photoreceptor defects compared with FAF

## Data Availability

Not applicable.
